# Serotonin-related Mechanisms in the Etiology and Pharmacotherapy of Social Phobia, A Review

**DOI:** 10.31661/gmj.v12i.3072

**Published:** 2023-12-18

**Authors:** Malihe Arjmandi Ghandashtani, Sahar Poudineh, Alireza Sarlak, Maryam Poudineh

**Affiliations:** ^1^ Psychiatry and Behavioral Sciences Research Center, Mashhad University of Medical Sciences, Mashhad, Iran; ^2^ School of Medicine, Mashhad Azad University, Mashhad, Iran; ^3^ Student Research Committee, School of Medicine, Hamadan University of Medical Sciences, Hamadan, Iran

**Keywords:** Social Phobia, Social Anxiety Disorder, Serotonin, Dopamine, Depression, Polymorphism

## Abstract

Social anxiety disorder (SAD), known as social phobia, is considered a prevalent psychiatric disorder characterized by a constant fear of social positions. Frequently, social phobia occurs with other mental disorders including depression and substance abuse conditions. Although SAD is considered one of the most common types of mental disorders, proper management may be compromised in recurrent psychiatric comorbidity due to clinicians’ focus on secondary complications. Moreover, despite the description of social phobia as a polygenic and complex condition, few altered genetic and epigenetic factors are identified as causative agents. Over the past decades, several studies have suggested polymorphisms in serotonergic and dopaminergic-related genes as the etiology of social phobia. Serotonin, on the other hand, as a necessary neurotransmitter in the central nervous system (CNS), is involved in a variety of disease processes including social phobia. Nevertheless, the exact mechanism of serotonin-dependent development of the disease and the efficacy of suggested pharmacotherapies are not fully understood. The current study aimed to review the serotonin-dependent mechanisms by which SAD develops and discuss the current suggested strategies that are based on serotonin metabolism.

## Introduction

Social anxiety disorder (SAD), also known as social phobia, is a prevalent psychiatric disorder [1]. A continuous and inordinate fear of social positions in which an individual may be subject to scrutiny by other persons is considered the most common characteristic of this disorder. The person with social phobia may fear being negatively evaluated including being judged as boring, weak, stupid, anxious, or unlovable [2]. Frequently, social phobia occurs with other mental disorders, the best examples of which are depression, avoidant personality disorder, and substance abuse conditions [3]. Moreover, SAD is associated with poor psycho-social functioning such as impaired workplace productivity, increased school drop-out, and lower socio-economic level [4][5].

SAD is considered to be one of the most common types of mental disorders with a lifetime prevalence of approximately 15% and a twelve-month prevalence of nearly 10% in adults. Moreover, a similar prevalence is reported among adolescents in the United States [6]. Social phobia has an early onset, the mean age is 13 years old and is well documented to be chronic in the majority of cases [7][8].

It is believed that symptoms including shame characterizing social phobia could hinder the proactive help request by patients with SAD [9]. In addition, proper management of SAD may be compromised in recurrent psychiatric comorbidity due to clinicians’ focus on secondary complications including alcoholism [10][11]. Nevertheless, functional solutions have been proposed, including the definition of biomarkers that can distinguish between these diseases, which may contribute to both correct diagnosis and selection of appropriate therapy, and also it could play a role in tracking patient status [12][13].

The promising biomarkers are described in different psychiatric disorders and include alterations in the genetic, epigenetic, structure, function of the brain, endocrine system, immune system, and neuropsychological aspects [13][14][15][16].It is widely accepted that social phobia is a polygenic and complex condition, however, few altered genetic and epigenetic factors are identified as causative agents. In addition, polymorphisms in serotonergic and dopaminergic-related genes are suggested to be associated with the emergence and development of social phobia [17].

Furthurmore, epigenetic alterations in serotonergic and dopaminergic-related genes including DNA methylation, described as environmental and genetic risk factors of social phobia, are attributed to SAD development [18]. Importantly, attributing stress to serotonergic and dopaminergic pathways, as well as introducing the etiology of the disease, can suggest diagnostic biomarkers and therapeutic strategies.

Currently, both psychotherapy and pharmacotherapy are considered to be the main strategies for social phobia treatment [19]. Cognitive behavioral therapy is known as the first-line treatment of SAD based on the National Institute for Health and Care Excellence (NICE) guidelines [20] which represent a response rate of 50% to 65% [21][22]. Regarding pharmacotherapeutic approaches, although benzodiazepines are suggested as initial or maybe as adjunctive treatment, particularly for patients requiring immediate symptom relief, selective serotonin uptake inhibitors (SSRIs) are widely recommended as first-line pharmacotherapeutics [23][24].

According to what was discussed above, the present study aimed to discuss the contribution of serotonin and its related factors in the pathoetiology of social phobia and the efficacy of serotonin-based therapeutic strategies, after an overview of serotonin biosynthesis and metabolism.

An Overview of Serotonin Biosynthesis and Metabolism

Serotonin (5-hydroxytrytamine) is considered an ancient indoleamine that is produced together with melatonin (N-acetyl-5-methoxytryptamine) [25][26]. The biosynthesis of serotonin by the first unicellular organisms formed on Earth should provide a means to cope with the oxygenated atmosphere after the evolution of photosynthetic organisms [27]. This ability of serotonin refers to its structure and its potential antioxidant property, which has been deeply studied today [28][29].

Serotonin was first reported in mammalian organisms in 1937, as the so-called enteramine, which was because of its presence in enterochromaffin cells of the gut inducing smooth muscle contraction [30]. The name serotonin was given to it later resulted of the identification and investigation by a separate lab in the 1940′s after finding the compounds to be the same [31]. Subsequently, further investigations led to the establishment of serotonin as a necessary neurotransmitter in the central nervous system (CNS) involved vitally in various disease processes, particularly in neurological disorders including depression, Alzheimer’s and Parkinson’s disease, and social phobia [32][33].

Two primary and distinct pathways, known as plant pathway and animal pathway, are described as mechanisms by which serotonin is produced. Both of these pathways are derived from the aromatic amino acid L-tryptophan. During the plant pathway the mentioned amino acid forms tryptamine by decarboxylation via tryptophan decarboxylase enzyme. After that, tryptamine is hydroxylated by tryptamine-5-hydroxylase resulting in serotonin formation [34]. In the animal pathway, the first two steps, carboxylation, and hydroxylation, are reversed with 5-hydroxytryptophan production by an enzyme called tryptophan hydroxylase, and 5-hydroxytryptophan is decarboxylated by aromatic acid decarboxylase enzyme to produce serotonin [35].

A plethora of evidence documented that the biosynthesis of serotonin is determined by the availability of its precursor tryptophan. The biosynthesis of tryptophan itself is from chorismate which is under almost tight feedback regulation by an anthranilate synthase enzyme [36]. In addition to being a precursor for serotonin production, tryptophan is considered a precursor for a variety of pivotal plant and animal metabolites [37][38].

Along with tryptophan and enzymes that convert this precursor to serotonin, enzymes that convert serotonin to other metabolites including melatonin have been introduced as determinants of serotonin concentration. N-acetyltransferase, for example, is responsible for the conversion of serotonin to N-acetylserotonin, which is a crucial regulatory step in the biosynthesis of melatonin via transcriptional control [39][40]

Serotonin-based Therapeutic Strategies

Serotonin exerts its biological effects as a neurotransmitter by binding to its receptors, which are defined as the largest family of G-protein coupled neurotransmitter receptors consisting of thirteen distinct genes encoding receptors of the G-protein coupled 7-transmembrane class. Along with those, another ligand-gated ion channel exists known as the 5-HT3 receptor [41][42]. The receptors of serotonin, which share significant orthology, are found in a remarkably diverse range of organisms from Caenorhabditis elegans and Drosophila melanogaster to humans. Because of this diversity, it has been contemplated that the primordial serotonin receptor from the rhodopsin-GPCR family probably first emerged more than 750 million years ago when predates the evolution of dopaminergic, muscarinic, and adrenergic receptor systems [43]. As a protein family, it is believed that G-protein coupled receptors to have evolved about 1.2 billion years ago [43]. Remarkably, the receptors of serotonin appear to be among the elder receptors of the rhodopsin-like family [44]. There are 3 main classes of G-protein-coupled 5-HT receptors including the 5-HT1A, 5-HT2, and 5-HT7-like receptors. It is documented that these receptors are less than 25% homologous and are differentiated approximately 700 million years ago, before the time that vertebrates diverged from invertebrates. Drosophila melanogaster expresses functional orthologs of the 5-HT1A, 5-HT2, and 5-HT7 receptors. Along with that, this fruit fly expresses orthologs for many other G-protein coupled receptors [45]. The mammalian 5-HT receptor subtypes have further differentiated over the last 90 million years.

Because of this long evolutionary history, serotonin plays a wide variety of roles in the native physiology including developmental, gastrointestinal, cardiovascular, and endocrine function, along with sensory perception and behaviors including sex, appetite, aggression, mood, cognition, sleep, and memory [41][42]. In mammals, the majority of serotonin is produced in the gut by the action of enterochromaffin cells. Moreover, serotonin could be reserved in blood platelets which are considered a relatively large pool of serotonin, allowing its isolation and structure clarification. Moreover, it is evidenced that serotonin exists in CNS demonstrating several varied but extremely vital functions. In mammals, CNS content of serotonin arises from the raphe nuclei, specialized groups of cell bodies located in the brainstem reticular formation [41][42]. As mentioned earlier, serotonin has antioxidant properties due to its unique structure, and in this sense, it plays an important role in maintaining health in different organisms [46][47].

In addition, serotonin has been defined as a neurotransmitter, thereby any related dysregulation associated with the function of serotonin or its receptors can be linked with neurological complications, and on the other hand, it can be used as a therapeutic strategy to cope with neurological disorders [48][49]. For example, schizophrenia, dementia, and cognitive impairment associated with Alzheimer’s disease (AD) have been targeted for clinically evaluated 5-HT6 receptor antagonists including idalopirdine, intepirdine, and latrepirdine [50].

Moreover, a selective 5-HT6 receptor antagonist known as masupirdine has been suggested to reduce aggression-like behaviors and psychosis in AD [50]. In addition, fluoxetine is considered a selective serotonin reuptake inhibitor capable of confronting cognitive disorders in patients with vascular dementia, depression, and AD [51][52]. In addition to these, research has also shown the role of serotonin in the development of AD and the role of serotonin 5-HT1A receptors in the modulation of depression, which confirms the effectiveness of related strategies in dealing with neurological disorders [53][54]. In this regard, the present study has aimed to discuss the therapeutic strategies dependent on serotonin and its receptors in the treatment of social phobia.

Serotonin-related Mechanisms in the Etiology and Treatment of SAD

Serotonin and related mechanisms have been suggested as the etiology of SAD; hence various studies have been conducted to counteract the disorder by manipulating serotonin-related pathways or to describe the serotonin-based etiology of the disorder to suggest novel therapeutic targets. In this regard, many studies have focused on the dysfunction of serotonin transporters to describe the pathogenesis of SAD as well as propose a therapeutic approach.

There is plenty of evidence that the human serotonin transporter (SERT), encoded by a single gene known as SLC6A4 located on the long arm of chromosome 17, could be involved in mental disorders. The serotonin transporter gene promoter region (5-HTTLPR) is considered the most investigated region of the SLC6A4 which is located 1kb upstream of the initiation site of SERT gene transcription. Because of the functional polymorphism consisting of a 44-base pair deletion/insertion in the 5’ regulatory area, 5-HTTLPR has recieved much attention. The long (L) allele is the so-called name of the variant with the 44-base pair insertion, without the 44-base pair whereas the variant is named the short (S) allele [1][2][3]. It is documented that the S variant of the 5-HTTLPR represents lower levels of SERT gene transcription [4][5].

As a result, lower levels of SERT transcription are associated with depression and other mental disorders. Miozzo et al. have revealed that among 628 participants with a mean age of 48.3 years old carriers of the S allele had a significantly increased risk of two specific comorbid disorder pairs which are major depressive disorder and social phobia [55]. Therefore, SERT promoter gene polymorphism may potentially play a role in identifying, subgrouping, and selecting treatment strategies. In addition, in a population-representative study consisting of 540 young adults and 453 adolescents, scientists revealed that the 5-HTTLPR genotype plays a key role in the association between phobia (fears) and perceived relations and a history of psychiatric disorder observed in participants who had S/S-genotype of 5-HTTLPR [56]. It is supposed that SAD entails an overactive presynaptic serotonergic system affecting the rate of serotonin biosynthesis and activity of tryptophan hydroxylase that, in turn, appears functionally influenced by the TPH2 G-703T polymorphism in the emotionally relevant regions of the brain [57][58].

In addition, several studies have investigated the role of SERT along with dopamine transporte (DAT) in SAD patients. A multi-tracer positron emission tomography study on 27 patients with SAD and 43 age- and sex-matched healthy subjects as controls revealed that social phobia was associated with remarkably increased expression and co-expression of the SERT and DAT in regions of the brain related to fear and reward [59]. Moreover, the resulting monoamine dysregulation is believed to underlie SAD symptomatology and constitutes a therapeutic target [59].

As a result, this evidence prompted researchers to investigate the function of serotonin and dopamine transporters as therapeutic targets. A randomized control trial on 27 participants including 17 men and 10 women administrated 9 consecutive weeks of overt or covert treatment with a selective serotonin reuptake inhibitor, escitalopram (20 mg). The findings revealed that SERT blockade alone was not sufficient for clinical response, however, anxiolytic effects of escitalopram treatment involve psychological factors contingent on dopaminergic neurotransmission [60]. Similarly, 9 weeks of treatment with escitalopram in 24 patients with social phobia demonstrated that the co-expression of SERT and DAT influenced the severity of symptoms and remission rate in the treatment of SAD [61]. Monoamine oxidase is an enzyme responsible for removing several neurotransmitters including serotonin, norepinephrine, and dopamine from the brain. Monoamine oxidase inhibitors (MAOIs) are among the first pharmacological treatments licensed for patients with neurological disorders including depression. In patients with SAD, a recent study revealed that the monoamine transporters are modulated in different ways when selective serotonin reuptake inhibitors or placebo is given concomitantly with cognitive-behavioral treatment [61].

It is believed that monoamine oxidase inhibitors could be beneficial in the pharmacological treatment of patients with SAD, thereby psychiatrists should be familiar with the pharmacological properties and potential uses of this pharmaceutics even though over time monoamine oxidase inhibitors were fallen out of the mainstream clinical application [62]. Recently, some guidelines have been published to describe how to use pharmacotherapies based on serotonin-dependent mechanisms in patients with SAD, which psychologists and psychiatrists vitally need to be familiar with [63][62].

In selecting treatment strategies, there is a pivotal necessity to consider variables including race, etiological beliefs, age, and gender of patients and the impact of these variables on treatment outcomes and potential adverse effects. It has been hypothesized that children with African American and Caucasian ancestors represent similar manifestations of anxiety disorder, however, the outcomes of treatment were different because of treatment barriers [64]. Genetic variations, especially in genes encoding enzymes and receptors related to serotonin, including bi- and triallelic SLC6A4 5-HTTLPR may contribute to the etiology of SAD and even affect how it responds to treatment [65].

Regarding the etiological hypothesis, it is believed that patients with "Need to be Liked" and "Bad Social Experiences" represent the most severe social anxiety manifestations, and patients with "Familial Factors" demonstrate the most rapid response to a selective serotonin reuptake inhibitor named paroxetine [66]. Interestingly, positive expectations towards the treatment process and hope for disease improvement have determined better results in treatment outcomes [67]. Fluvoxamine is considered one of the selective serotonin reuptake inhibitors and is recommended as the primary treatment for several neurological disorders including anxiety disorders, social phobia, panic disorder, and post-traumatic stress disorder [68].

However, several adverse effects including male infertility (due to deleterious effects on spermiogram and steroid hormones) and hematologic dysfunctions (e.g. lymphocytosis, leukocytosis, and monocytosis) have been reported [69]. Escitalopram is another type of selective serotonin reuptake inhibitor which is widely prescribed to treat depression and anxiety disorders. In a randomized controlled trial on 46 patients with SAD, researchers found that the administration of escitalopram caused no serious adverse effects [69]. Furthermore, it has been suggested that the treatment of severe cases of SAD requires the use of selective serotonin reuptake inhibitors combined with cognitive behavioral therapy rather monotherapy [70]. Therefore, it is necessary to choose the best treatment method according to the mentioned variables along with the clinical manifestations.

## Conclusion

The current study revealed that polymorphisms in the genes encoding serotonin and dopamine transporters are mainly involved in the development of social phobia. Moreover, serotonin-dependent therapeutics including selective serotonin reuptake inhibitors and monoamine oxidase inhibitors could be considered pharmacotherapeutics in the treatment of patients with SAD. However, limitations including adverse effects, monotherapy insufficiency, and first-line therapy selection require further studies.

## Conflict of Interest

None.
